# Applying data mining techniques to medical time series: an empirical case study in electroencephalography and stabilometry

**DOI:** 10.1016/j.csbj.2016.05.002

**Published:** 2016-05-18

**Authors:** A. Anguera, J.M. Barreiro, J.A. Lara, D. Lizcano

**Affiliations:** a Technical University of Madrid, School of Computer Science, Campus de Montegancedo, s/n - 28660, Boadilla del Monte, Madrid, Spain; b Open University of Madrid, UDIMA - Facultad de Enseñanzas Técnicas, Ctra. De la Coruña, km 38.500 – Vía de Servicio, 15 - 28400, Collado Villalba, Madrid, Spain

**Keywords:** Medical Data Mining, Electronic Health Record, Time Series, Knowledge Discovery

## Abstract

One of the major challenges in the medical domain today is how to exploit the huge amount of data that this field generates. To do this, approaches are required that are capable of discovering knowledge that is useful for decision making in the medical field. Time series are data types that are common in the medical domain and require specialized analysis techniques and tools, especially if the information of interest to specialists is concentrated within particular time series regions, known as events.

This research followed the steps specified by the so-called knowledge discovery in databases (KDD) process to discover knowledge from medical time series derived from stabilometric (396 series) and electroencephalographic (200) patient electronic health records (EHR). The view offered in the paper is based on the experience gathered as part of the VIIP project.^1^

Knowledge discovery in medical time series has a number of difficulties and implications that are highlighted by illustrating the application of several techniques that cover the entire KDD process through two case studies.

This paper illustrates the application of different knowledge discovery techniques for the purposes of classification within the above domains. The accuracy of this application for the two classes considered in each case is 99.86% and 98.11% for epilepsy diagnosis in the electroencephalography (EEG) domain and 99.4% and 99.1% for early-age sports talent classification in the stabilometry domain. The KDD techniques achieve better results than other traditional neural network-based classification techniques.

## Highlights

• Time series are data types that are common in the medical domain.
• The application of data mining in medical time series has many implications.
• We presented a CDS system built based on medical time series from patient EHRs.
• We discovered useful knowledge from medical time series derived from stabilometric and EEG patient EHRs.
• This paper offers guidance on how to address data mining tasks in medical time series.

## 1 Introduction

The quantity of information generated by the many different activities carried out in medicine is constantly on the increase. The efficient and responsible use of this information is one of the key challenges today.

In the healthcare field, information is generated at many different levels: management, planning, medical examinations, etc. In particular, the research described in this paper focuses on patient medical data, formally known as electronic health records (EHR).

EHRs may contain very wide-ranging data types: nominal (ICD9 codes, CPT codes), ordinal (pain scales, PEW scores), numerical (temperature, BP), unstructured clinical narratives (for which text mining techniques are required), etc. There is a lot of literature on clinical systems operating on these data types [1]. However, more and more EHRs contain a data type whose structure may, on occasions, be extremely complex and which has been found after investigation not to have been thoroughly researched: time series.

A time series can be defined as a sequence *TS* of time-ordered data *TS* = {*TS*_*t*_, *t* = 1,…,*N*}, where *t* represents time, *N* is the number of observations made during that time period and *TS*_*t*_ is the value measured at time instant *t*. The results of medical examinations (electroencephalogram, electrocardiogram, electromyogram, etc.) very often constitute a time series [2], [3]. Such is the importance of time series in medicine today that important data types like medical images (radiodiagnosis) are also very often mapped as time series for later processing and analysis [4].

The analysis of time series for knowledge discovery is far from straightforward and requires the application of special-purpose tools, especially if the key information of interest to the expert is concentrated within particular time series regions, known as events. Data mining is an interesting option in this respect. As illustrated by the success stories described by Shadabi and Sharma [5], data mining techniques have a huge potential for analysing such large volumes of stored medical data in order to discover knowledge. Generally, the extraction of useful, tacit and previously unknown knowledge from large data volumes is what is known as knowledge discovery in databases (KDD). The KDD process ranges from the understanding and preparation of the data to the interpretation and use of the discovered knowledge (results of the KDD process). Data mining is the stage of the KDD process where the data are studied and useful information is extracted using a set of techniques and tools [6].

Traditional time series analysis techniques examine whole time series. However, the techniques applied in this case study were especially designed to address the analysis of time series events. As discussed in detail later, together, these techniques solve a classification problem, for example, by means of a strategy combining:

a) The identification of time series events
b) The generation of time series reference models for several subjects
c) The comparison of a subject (to be classified) with different reference models.

The aim of this paper is to report the results of two case studies applying the above techniques and also share with the scientific community the experience that we have gained in the field of medical time series analysis, highlighting the particularities of medical time series processing throughout the different stages of the KDD process. To do this the case study research methodology was used in order to propose and apply advanced knowledge discovery techniques on data from two branches of medicine: stabilometry and electroencephalography. In doing so, the above process was supervised in its entirety by medical specialists from the respective fields. A sample of their impressions is reported as lessons learned in Section 5. Other researchers may find the experience shared in this paper useful for more efficiently and successfully undertaking similar projects for extracting useful knowledge from other medical time series.

The remainder of the paper is organized as follows. Section 2 discusses some papers and concepts of interest related to our proposal. Section 3 describes the reference domains used in this research. Section 4 details the process enacted to extract knowledge from time series, as well as the results of its application. Section 5 briefly discusses different issues of interest related to the proposed techniques and the illustrated case study (applicability, relationship to other techniques, limitations, viewpoint of medical experts, etc.). Finally, Section 6 reports the conclusions of the research and states some challenges in this field.

## 2 Background

The literature covers different approaches based on the application of computer techniques applied to the domain of medicine. Some are based exclusively on expert knowledge [7], [8], [9], [10], [11], [12]. Others, however, learn from previous problems (case-based approaches) [13], [14] or are representations (e.g., decision trees) that support decision making (model-based approaches) [15]. There are also hybrid approaches, such as the one illustrated in this article, where expert knowledge is used to gain a better understanding of the domain and KDD techniques are then applied to build models for use in decision making (e.g., diagnosis) based on the medical data.

The KDD process includes the following stages (which may vary slightly from author to author) [6]:

1. **Domain and data understanding**. This first phase (which some authors consider to be outside the scope of the KDD process) studies the general characteristics of the data to be analysed and the source domain.
2. **Data selection**. This phase determines all the sources of data of interest, which are unified in a target dataset.
3. **Data preprocessing**. The goal of this stage is to assure the quality of the data. To do this, a series of tasks are performed on the dataset generated in the selection phase. These tasks include reducing noise, handling missing values, etc.
4. **Data transformation and reduction**. In this phase, the preprocessed data are subjected to a number of filters and operations in order to assure that the data format is suitable for running data mining algorithms.
5. **Data mining**. A series of techniques and machine learning algorithms can be applied to the correctly formatted data in order to discover knowledge. These techniques are applied in order to solve different problem types, known as tasks.
6. **Knowledge interpretation/evaluation**. The last step in the KDD process aims to evaluate the resulting models and, if the assessment is positive, interpret the knowledge inferred from the models.

Clearly, KDD is a well-established process divided into phases and tasks. It generally functions as a paradigmatic framework for discovering knowledge from the data of any domain. And medicine is not immune either to the beneficial effects of being able to access a highly standardized and widely documented framework such as the above. In fact, applying the KDD process to a branch of medicine by documenting and storing (whenever possible) the interim and final results could be a major step forward in medical research based on data analysis.

### 2.1 Time series analysis techniques

There are a great many techniques related to time series analysis in the literature.

There are techniques for comparing time series and extracting common subsequences. The most noteworthy are techniques based on Fourier [6] or wavelet [16], [17], [18] transforms. Others are based on comparing time series singularities, known as landmarks [19]. Unlike the above, another group of techniques address the time series directly, using concepts such as the time warping distance [20], [21], minimum bounding rectangles (MBR) [22], Markovian models [23] or graph theory [24]. Of the above, the wavelet-based technique is most closely related to our proposal, as it is somehow capable of identifying events. The drawback of this technique, however, is that the events in question (wavelets) do not necessarily match up with the segments of interest to domain experts. The other techniques described in this section are useful for comparing two whole time series. These techniques apply different methods to extract information on the entire time series. In many domains, like EEG or stabilometry, the focus should be exclusively on regions of interest (events) in the time series.

There are techniques not only for comparison but also for generating transform-based reference models [25]. Again, however, they analyse the whole time series in order to output the transform coefficients (which are modelled). The same applies to other research aiming to find parts that a group of time series have in common but which are not necessarily of interest to the specialist [26], [27], [28], [29]. Some techniques are based on previously transforming the series into a set of segments. Even so, their applicability is confined to specified domains [30].

On the other hand, there are some proposals in the literature related to event identification. They are linked to specified domains, which means that they are either not usually generally applicable [31], [32] or are based on identifying the prominently shaped segments of the series [33], [34], [35] that do not necessarily match up with the events that are of interest to domain specialists.

Finally, this article illustrates an example of time series classification. Note, therefore, that most of the reviewed literature concerns traditional techniques like the simple nearest neighbour algorithm [36], [37], [38]. We have also found techniques that are more like the approach reported here and are based on distinctively identifying subsequences in time series (not necessarily events of interest for experts) [39].

### 2.2 Time series analysis techniques applied to medicine

Other authors have proposed different approaches to time series analysis techniques for the medical domain. Firstly, several authors conducted an interesting survey of time series with regard to what they are capable of modelling and why they should be used to analyse the complexity of some multidimensional data that would otherwise not be understandable for expert systems analysing raw data [40]. These time series have been widely used in the field of KDD applied to medicine in many papers. A prominent example is the data mining research applied to the classification and treatment of known medical conditions [41], proposing an expert system that classifies and automatically recommends a treatment based on a history of known diseases and disorders (partly characterized by data mining-based measurements and diagnoses). Another similar and again very interesting article [42] proposes a time series clustering system based on formal concept analysis. This prototype outperformed other classical clustering techniques, although it had a problem in common with many other proposals of this type: medical specialists find it hard to select the right techniques, tools, steps and technologies in order to undertake KDD. On this ground, several authors put together a practical guide for the above KDD-focused phases in the field of medicine [43]. Finally, another problem with respect to time series management in a domain as complex as medicine is the explosion of complexity resulting in multi-valued data clustering tasks. On this ground, many papers are in favour of reducing data dimensionality by abstracting subseries of interest. This would simplify the data analysis and classification processes. [44].

In any case, and as we are given to understand by the above and papers like [45], where several authors review the state of the art up until 2011 on clustering and other implemented techniques for KDD from time series, this is a key data type in medicine today. Hence, there is a need for proposals like the one outlined in this article, whose aim is to communicate a case study on time series events analysis, an issue not previously addressed in the medical field by any of the above techniques.

## 3 Reference Domains: EEG And Stabilometry

### 3.1 The EEG field

Electroencephalography (EEG) is a branch of medicine responsible for studying electrical brain activity. To do this, it uses an electroencephalogram machine, which is able to graphically represent this activity. Electroencephalography is used among other things to diagnose disorders like epilepsy and brain injuries or tumours. The signals generated by an electroencephalogram are time series, whose analysis has brought major advances in the medical domain [46], [47], [48].

In the past, electroencephalography was a tool used exclusively by physicians. Recently, different methods from the intelligent systems field have been applied to discover knowledge from electroencephalographic time series [49], [50]. This was the perfect opportunity to specify medical knowledge and standardize different diagnostic procedures.

Electroencephalographic devices generate time series that record electrical activity (voltage) generated by brain structures over the scalp. EEG signals contain a series of waves characterized by their frequency and amplitude. EEG time series include certain types of special waves that are characteristic of some neurological pathologies, like epilepsy. Such waves are known as paroxysmal abnormalities and can be considered as events (special regions of the time series that are interesting for domain experts).

**Fig. 1:**
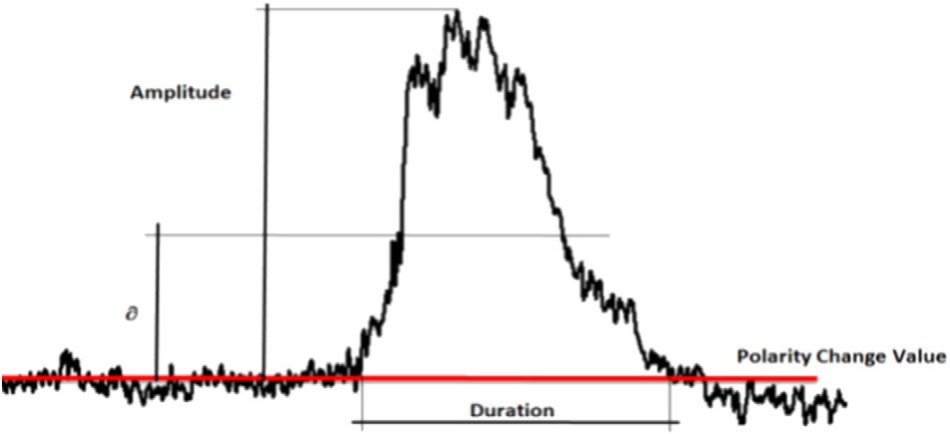
Event taken from an EEG time series.

During this research we have taken into account three kinds of events:

• Spike wave: A wave whose amplitude is relatively higher than the other waves in the signal and has a period of between 20 and 70 milliseconds.
• Sharp wave: A wave whose amplitude is relatively higher than the other waves in the signal and has a period of between 70 and 200 milliseconds (see Fig. 1).
• Spicule: A sharp wave with an abrupt change of polarity.

The features characterizing these events are the duration and amplitude of the wave, as shown in Fig. 1.

### 3.2 Stabilometry Field

Stabilometry is a branch of medicine responsible for studying human postural control [51], [52]. Postural control is a key element for understanding a person's ability to perform their routine activities.

**Fig. 2:**
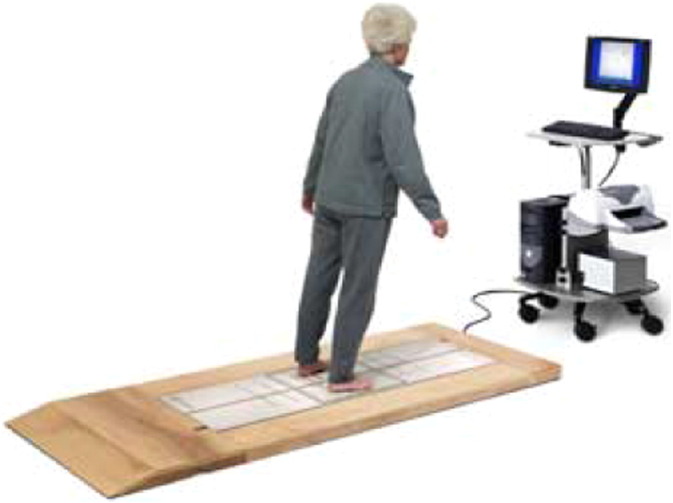
Patient performing a test on a stabilometric platform.

Postural control is measured by means of a device called a posturograph. To do this, patients take a series of tests, designed to single out the major sensory, motor and biomechanical components that contribute to people's balance [53]. Fig. 2 shows a patient performing a posturographic test.

Although stabilometry was originally devised merely as a technique for assessing a patient's postural control and balance, it is now considered to be a useful tool for diagnosing and treating balance-related disorders [54], [55], [56], [57], [58], [59].

Throughout this research, we have used a posturography device called Balance Master, manufactured by NeuroCom® International [60]. The device is composed of a metal plate placed on the floor and divided into two interconnected longitudinal plates. The metal plate is surrounded by a wooden platform, whose sole mission is to prevent patients from stumbling and falling. The patient stands on the metal plate and completes different types of tests, called US, LOS, BIS, RWS and WBS [61]:

• US (*Unilateral Stance*): The aim of this assessment protocol is to measure the ability of patients to keep their balance standing on one foot with either eyes open or eyes closed.
• LOS (*Limits of Stability*): The aim of this assessment protocol is to measure the maximum distance that patients with both feet on the platform can intentionally displace their centre of gravity without losing balance or stepping for a time.
• BIS (*Bilateral Stance*): The aim of this assessment protocol is measure patient balance on different surface types during which patients must stand still on the platform on top of first a firm surface and then a foam surface.
• RWS (*Rhythmic Weight Shift*): The aim of this assessment protocol is to quantify patient ability to voluntarily move their centre of gravity laterally from left to right and forward and backward between two targets at various speeds.
• WBS (*Weight Bearing Squat*):The aim of this assessment protocol is to measure the percentage of body weight borne by each of the two legs. Ideally each leg should bear approximately half the body weight.

**Fig. 3:**
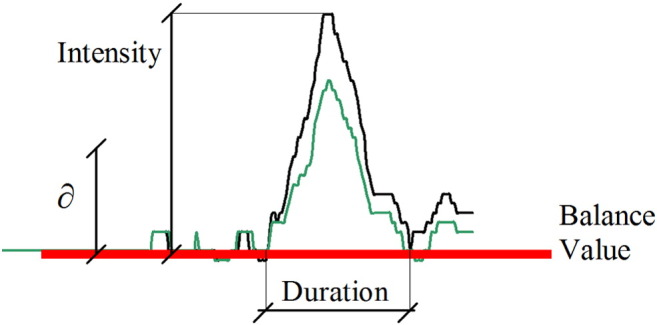
Fall event taken from a stabilometric time series.

These tests generate time series that measure patient balance. This case study focused on the US test, as, according to the consulted experts, this is the assessment protocol that reveals most information about subject balance. The aim of the US test is to measure how well able patients are to keep their balance standing on one foot with either eyes open or eyes closed. Ideally patients should remain perfectly static with no sway throughout the test. An interesting event type for this test is located at times when patients lose their balance and put their raised foot down on the platform. This event type is known in the domain as a *fall* and is identified when the pressure on the sensor corresponding to the lifted leg is greater than a specified threshold (∂). These events are characterized by their duration and intensity (see Fig. 3).

## 4 Applied Methods and Results

The case study reported below stated two different scenarios, one for each of the two reference domains. The KDD process was enacted from start to finish in each of the above scenarios, applying the specified techniques (see 4.1, 4.2, 4.3, 4.4, 4.5, 4.6). The ultimate aim was to classify individuals represented by their respective time series. In fact, two reference models were output for each domain (healthy and epileptic for EEG; basketball players and ice-skaters for stabilometry). The idea was to test the discriminatory power of the proposed classification strategy, adopting for this purpose the approach defined by the cross-validation technique (90% for training; 10% for testing).

In the statistical study conducted throughout the case study, tried and tested descriptive and predictive methods were applied for hypothesis testing based on p-values whose critical value was consistently below the confidence threshold of α = 0.05.

The entire process is described below.

### 4.1 Understanding the domain and the data

The first step was to understand the data for each of the two domains.

To do this, time series first had to be studied thoroughly, analysing their many features. Some of the key characteristics are:

• Size of the time series (number of timestamps). Size can determine the amount of resources required to store and process the series. In this case, the size of the time series is manageable (from 1000 to 4000 timestamps).
• Type of recorded value. Values will generally be numerical, as applies to this research. This generally makes the research simpler, as there is a wider range of techniques for use or from which to borrow ideas.
• Regions of special interest. Time series may have regions that are of interest to domain experts. These regions have to be identified and characterized. Such regions are known as **events** and are usually a very common feature of medical time series. It is evident in this case that there are events of interest, as discussed in Section 4.3.
• Regions without interest. If there are regions of special interest (events), the other regions may be of less or even of no interest. It is important to clarify this point with experts. In this research, the experts specified that time series regions that were not events could be disregarded.
• Range of recorded values. The value range is necessary in order to identify any regions of interest.
• Distance between measurements of the time series, paying special attention to whether or not there is a pattern. This was 10 milliseconds in the time series used in this research.
• Possible noise in the series. Noise may be caused by many factors. The identification of noise and the respective factors will help to correct or minimize noise. As discussed in Section 4.3, most noise is caused by the patient and the expert supervising the test being out of phase.

Interaction with experts would appear to be crucial for dealing with the above questions, etc. Experience suggests that it is highly advisable to consult a group or panel of experts for multiple gold-standard annotation rather than relying on a single expert [61], [62], [63], [64], [65], [66], [67], [68]. The premises of this panel-based approach are as follows:

1. There are two or more individuals, each characterized by his or her own perceptions, attitudes, motivations, and personalities,
2. who recognize the existence of a common problem, and
3. attempt to reach a collective decision.

A panel of experts often participates in different decision-making rounds. The decisions made by each particular member are used as input for new decision-making rounds involving the whole panel. The Delphi method is an example of an expert panel technique. Using techniques like this, experts have access to the decisions of their peers. This can lead them to change or add to the decisions that they made based on the viewpoints of other experts [69]. In this research, the Delphi method was used for all expert consultations (the panel was composed of five experts), and consensus was reached in two or three rounds depending on the task in question.

#### 4.1.1 Conceptual modelling

Apart from the above, the domain and data may be easier to understand using conceptual modelling mechanisms. The conceptual modelling of a dataset has many benefits:

■ It is useful for clearly establishing relationships among different dataset entities, especially when the dataset contains different levels or hierarchies.
■ It is useful for representing the entity attributes, as well as the possible attribute value types.
■ A visual data representation is useful for giving a rapid and intuitive overview of the dataset.
■ Conceptual modelling is often the basis for later data storage in databases.
■ Additionally, conceptual modelling is the potential starting point for automating other tasks such as the comparison of individuals or the generation of reference models.
■ Modelling specifies and standardizes data and is the starting point for their transformation to other models of different levels of abstraction [70].

When conceptually modelling the reference domains (and other areas), it was found that, in all the studied cases, there is a central entity or *register* that represents the analysed object (in this case, a patient). Other lower-level data entities including different *measurements* of the object under analysis (for example, a patient EEG) usually depend on the central entity. Some *conditions* are usually altered when these measurements are taken in order to check the behaviour with different parameters (for example, an EEG of an epileptic patient could be repeated immediately after a seizure or a long time after the last seizure). The data collected from each of the measurements under each particular condition may be single valued or adopt more complex structures, like, for example, time series. Data engineers that undertake a project in the field of medicine must be aware that they will come across large volumes of complex, high-dimensional data types. For example, patient stabilometric data are composed of several tens of time series and several tens of single-valued attributes, and a patient's stabilometric data total around three megabytes of information.

**Fig. 4:**
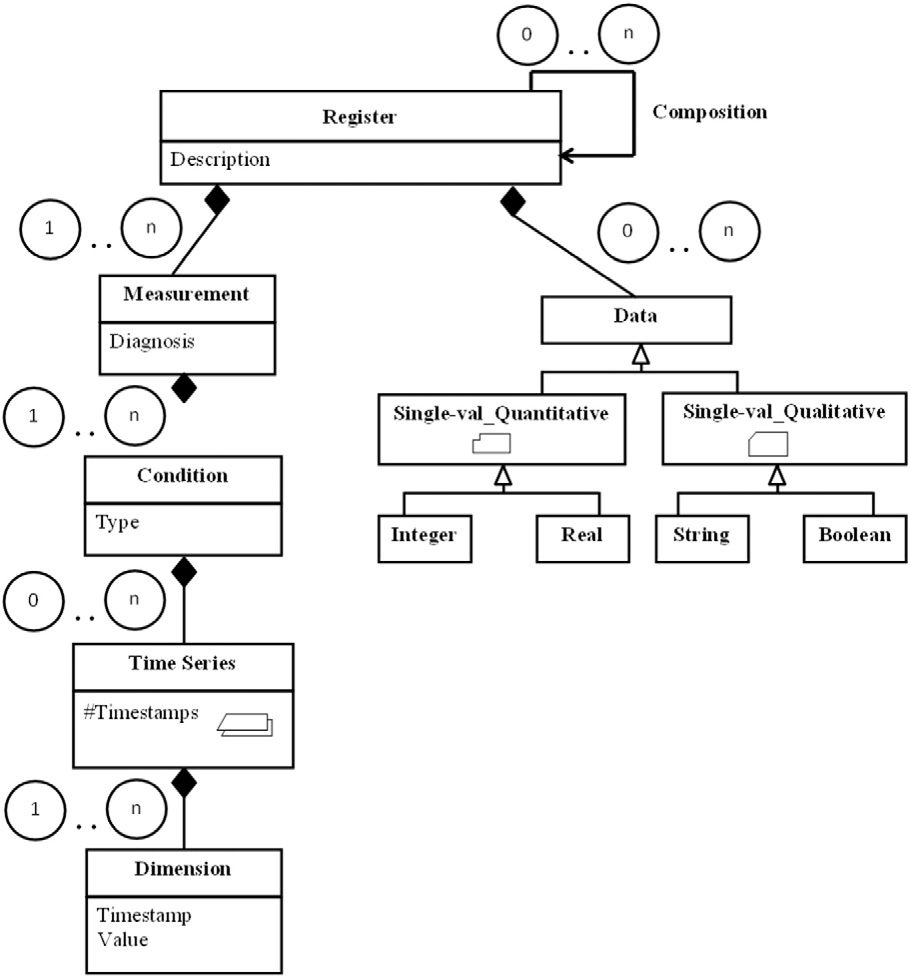
Generic UML model.

Following the above structure, common to any branch of medicine, a general-purpose procedure was proposed for conceptually modelling data in UML2, as illustrated in Fig. 4. This generic model is able to automate the medical data preprocessing phase. The proposed model includes stereotypes, a mechanism for extending UML2 whereby it is endowed with more meaningful conceptual representations using icons and constraints based on a UML mechanism called *profile*. For an exhaustive description of the above stereotypes, see [65].

Fig. 4 highlights the above concepts of *register*, *measurement* and *condition.* It also shows all the possible data types that may condition the data mining techniques: *time series* are processed differently to *single-valued* data, which are, in turn, often processed differently depending on whether they are *quantitative* or *qualitative*. Note that the above concepts are organized hierarchically in the form of a tree, where register is the root and the times series and single-valued data (represented by *data*) are the leaves.

**Fig. 5:**
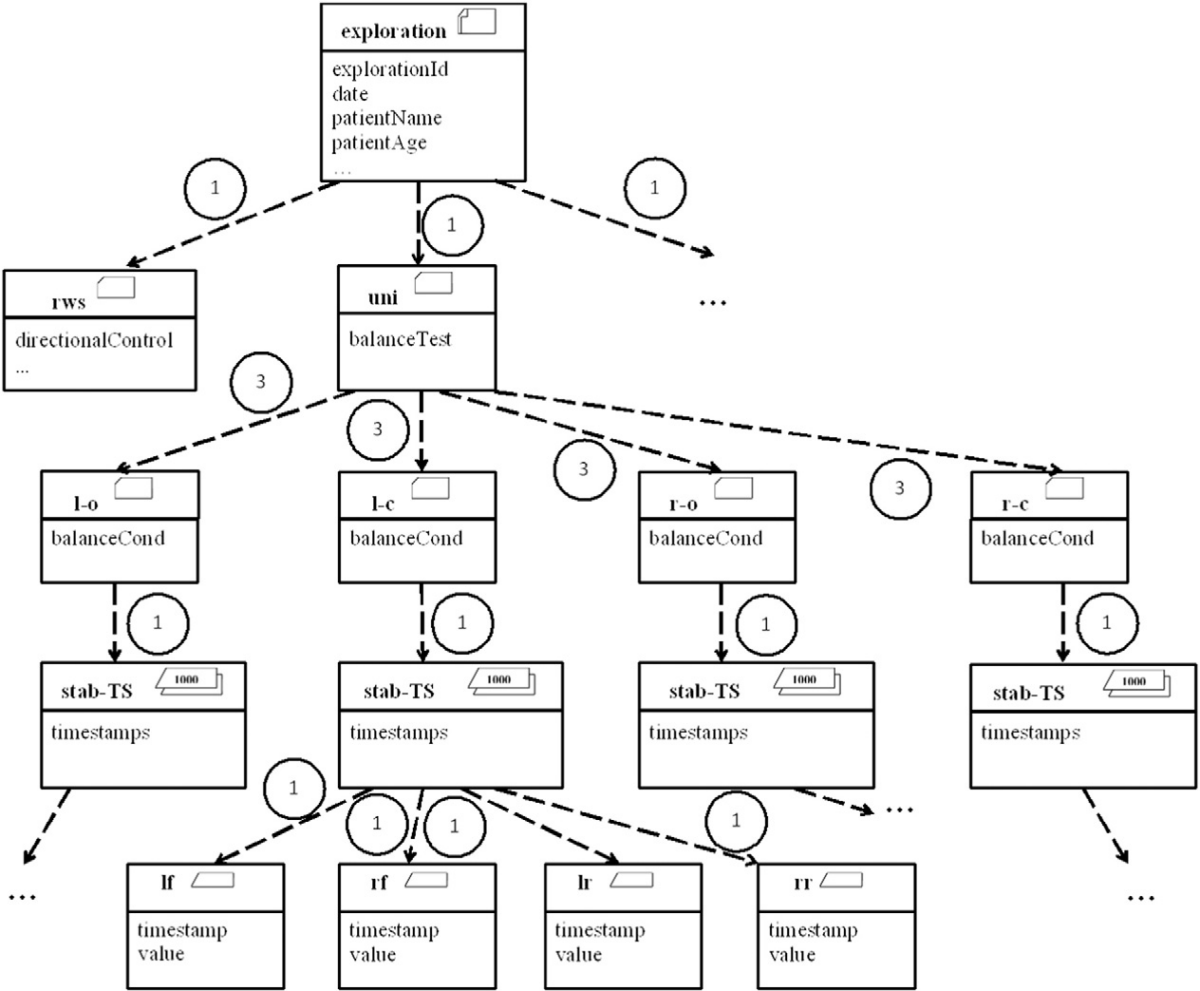
Part of the conceptual model of the stabilometry domain [65].

The above generic notation has to be tailored to each domain of experimentation. For example, Fig. 5 shows the model tailored for stabilometry domain data.

The proposed notation has been used as a major support tool for understanding the analysed data and domains, as well as reducing the workload necessary for developing the other tasks. As reported by Lara et al. [65], the domain and data understanding phase can be performed about 1.6 times faster using the proposed notation in the studied domains.

Apart from this advantage, the data gathered from each subject have been stored according to this conceptual model. Additionally, each individual data model is later used in data mining techniques to provide the structure guiding the different algorithms.

### 4.2 Data selection

Several electroencephalographic and stabilometric data sources were used throughout this research.

With respect to the electroencephalographic domain, the publicly available data described by Andrzejak et al. [71] were used. They include data from real patients. The complete dataset consists of five sets (denoted A–E), each containing 100 single-channel EEG segments. These segments were selected and cut out from continuous multi-channel EEG recordings after visual inspection for artefacts, e.g., due to muscle activity or eye movements. Sets A and B consisted of segments taken from surface EEG recordings that were carried out on five healthy volunteers. Volunteers were relaxed in an awake state with eyes open (A) and eyes closed (B), respectively. Sets C, D, and E originated from an EEG archive of presurgical diagnosis. The case study reported in this paper focused on sets A and E. The data source only reports patient examinations and does not include any demographic information about the subjects. Note that the patient partitioning into the subsets was determined by the original dataset, and this division was not performed for the purposes of cross-validating the potential classification methods to be applied.

As regards the stabilometry domain, we used data from real top athletes, including professional basketball players and elite ice-skaters. The study was conducted on young, white males (practising professional athletes).

**Fig. 6:**
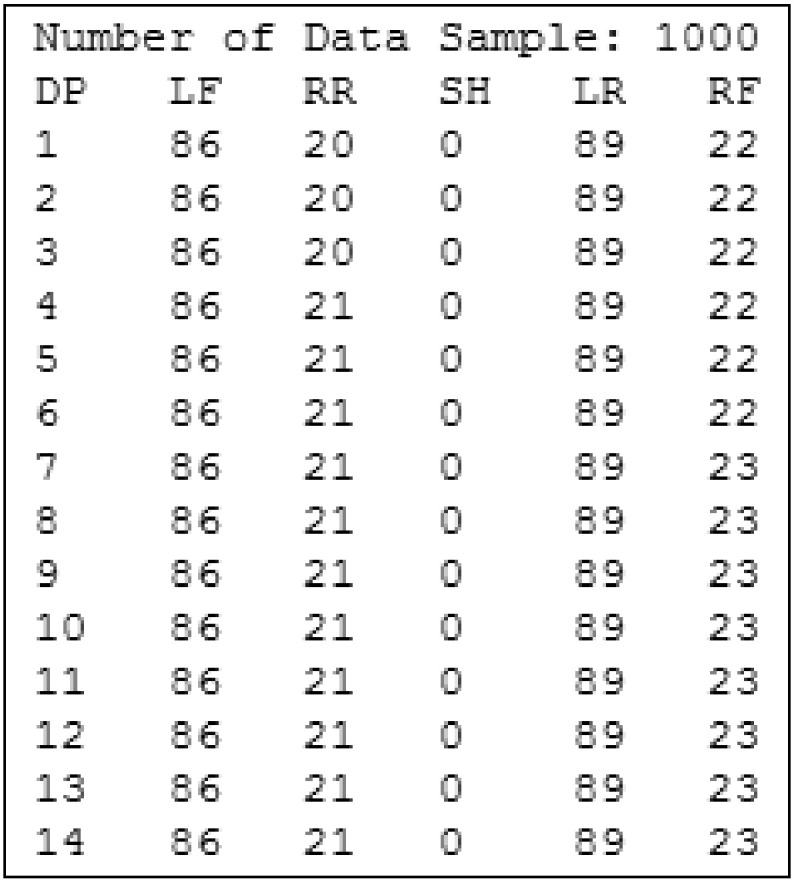
Stabilometric time series snippet.

The input data associated with subjects were, in both cases, first and foremost time series generated after medical examinations. These time series were composed of numerical values generated on the spot during medical check-ups and stored in plain text files. As illustrated in Section 4.3, these files are converted into XML documents which can then be automatically preprocessed. In both reference domains, time series size is defined by the number of observations. This value is equal to 1000 in the stabilometric domain and 4000 in the EEG domain. The sampling period was 10 milliseconds. Fig. 6 shows a time series snippet for the stabilometry domain.

**Fig. 7:**
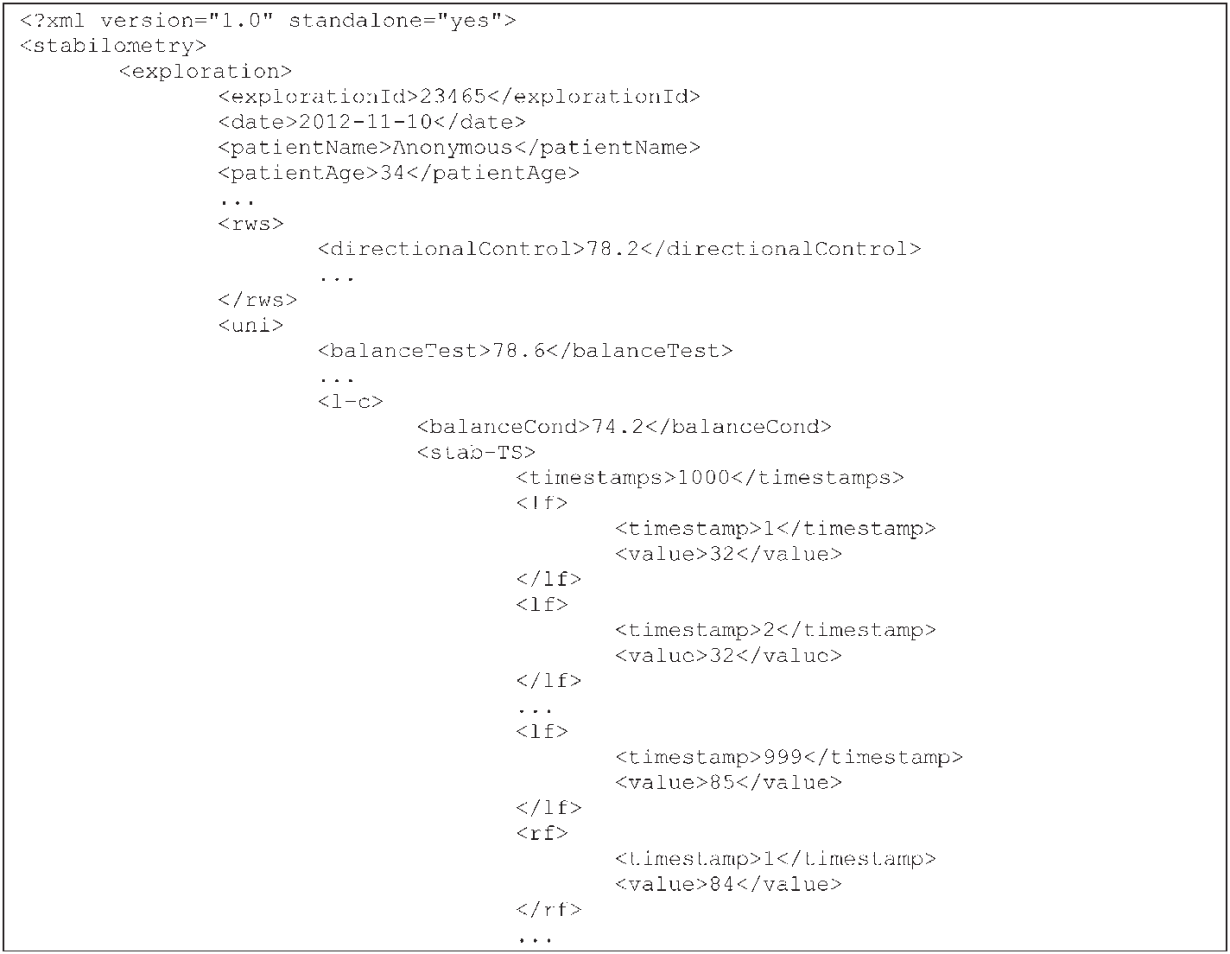
XML snippet containing patient stabilometric data.

These data have to be stored in a repository. Since this was a small project, XML documents were used to store the time series in conventional databases. Fig. 7 shows a snippet of an XML document generated from a patient profile in the field of stabilometry, one of the reference domains used in this research. This and other similar documents were used as a data source from which to extract useful knowledge.

Clearly, this is a pseudo ad hoc extract, transform and load (ETL) process, whereby information from medical tests are dumped, offline, in an information repository based on standard XML. The schema of these XML documents does in fact conform to the conceptual modelling pattern (for example, Fig. 5).

However, this proposal was found to have some weaknesses with respect to flexibility and efficiency as bigger data were processed. In this respect, the use of big data methods (based on efficient distributed information storage frameworks) and open standards (such as HL7 [72] or i2b2 [73]) could be a major advantage.

### 4.3 Data preprocessing

At this stage it is crucial to address noise and missing values. In this case, both circumstances were found to be the result of the patient and the test supervisor being out of phase with respect to the start and end of the test. This meant that there was noise and missing data at the start and end of the time series. These fragments were eliminated so that the time series only contained the parts that were really consistent with the examination. In actual fact, the physician supervising the test is responsible for cancelling the test if time series noise is not only confined to the beginning and end of the times series but also affects a considerable part of the remainder of the series. As a result, the first filter is applied manually.

Additionally, the same automatic strategy was enacted with respect to noise management and missing values:

1. Omit the missing values (pressure equal to 0 recorded by the respective sensor) or inconsistent values (according to established domain-dependent thresholds).
2. If at least 70% of the values can be retrieved after step 1 above, the time series is considered to be valid.
3. Otherwise, the time series is omitted from the respective data modelling tree and is not considered for comparison and conceptual modelling.

Apart from the above, a mechanism, based on the generic conceptual model common to both analysed fields (Fig. 4), was devised for automatically transforming the data of any medical field to an equivalent format on which data mining techniques can operate directly. To be precise, a standard and well-known target XML schema definition (XSD) was defined, in conjunction with an automatic mechanism for transforming an XML that does not fully conform to the above schema into another equivalent and fully compliant XML, applying for this purpose finite and non-ambiguous XSLT transformations [36]. As discussed in Section 4.2, the availability of XML data sources (see example in Fig. 7) is useful for quickly inferring the domain data structure and using automated mechanisms such as this during the data processing phase.

The architecture supporting this automatic data preparation mechanism uses the proposed UML2 model, which is mapped to description logic by means of a series of XSLT-based transformations, a target XSD and a source XML schema. A tool called eMOFLON [76] is used to automatically build a rule box called ABox, “AssertionComponent”, from the output description logic. The description logic is also used to build a terms box called TBox, “TerminologicalComponent”, which contains a description of the terms used (*register*, *measurement*, *condition*, etc.). In this manner, the eMOFLON tool is capable of mathematically describing the domain schema and XML format used in the data from the user-defined descriptive logic. The outputs of executing this tool (the above ABox and TBox) feed another tool, called RACER, whose input is the XML data of any domain and their respective XSD. RACER outputs two Boolean values: subsumption and instance. Subsumption indicates whether the input component model is a subsumption of the generic model, and instance indicates whether the component syntax is an instance of the generic model and a new ABox’ component that contains a series of XSLT mappings. The XSLT mappings are applied to the source XML data and XSD and transform the data into other equivalent data structured to conform to the proposed generic UML2 model. In other words, RACER is capable of calculating a set of XSLT mappings that can modify an XML whose structure does not conform to a XSD in order to make an equivalent XSD-compliant XML for the above descriptive logic.

The automatic data preprocessing mechanism is capable, according to experiments, of reducing the error rate in the preprocessing phase to at most 2%. Besides the low error rate, automatic preprocessing saves time and effort. In any case, the time taken to apply the proposed mechanism is, according to the results, linearly correlated to the size (number of lines) of the generated XML and XSD data files of 0.99 and 0.56, respectively. This linear behaviour evidences the scalability of our proposal.

### 4.4 Data transformation and reduction

After preprocessing the data automatically, it is necessary in this proposal to apply filters in order, for example, to reduce data dimensionality.

The main filter for reducing data applied in this proposal is time series event identification, applicable if only some parts rather than the whole time series are of interest. The identification of events in times series is a complex task and requires costly ad hoc methods for each domain. Therefore, we proposed the **time series event definition language**[67]. This language enables domain experts to simply and naturally define any events appearing in the time series of each domain.

**Fig. 8:**
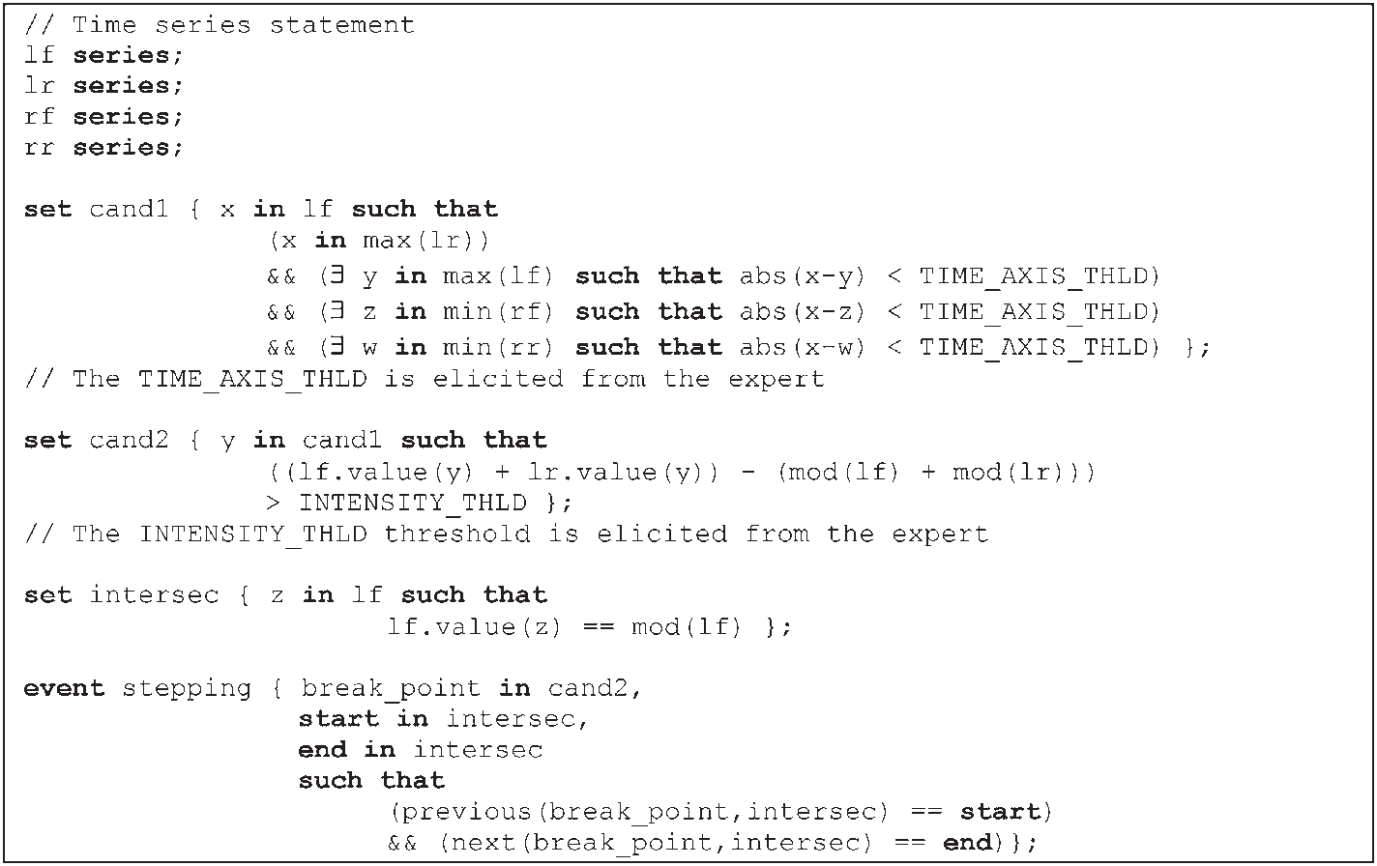
Definition of events for the US stabilometric test.

For example, Fig. 8 shows an excerpt from the event definition process for one of the stabilometric domain tests. The notation proposed by time series event definition language was used for this purpose.

After applying the event identification technique, each series was mapped to a set of events, each characterized by a series of all numerical characteristics. These are the event-related features that were described in 3.1, 3.2 (see Fig. 1, Fig. 3). They will be the input data source for the data mining algorithms.

The results of applying the above technique are reported below.

• EEG

This experiment focused on sets *A* (healthy patients with open eyes) and *E* (epileptic patients during an episode). It is precisely the wealth of these data and their availability that led us to explore this medical domain in order to validate the proposed model. First, we applied the event definition language in order to discover events from a total of more than 200 time series. In order to evaluate the accuracy of our event identification proposal, a number of EEG domain experts were asked to identify the events in the above 200 time series. The proposed technique was then applied to do the same thing. The accuracy of the proposal was calculated according to Eq. (1) which measures the match between the events specified by the experts and identified by the proposed language for all time series. In Eq. (1), #*Ev*_*Lang*_ stands for the number of events identified by the language, #*Ev*_*Exp*_ is the number of events specified by the experts and #*Ev*_*Lang-Exp*_ stands for the number of events detected by the experts that were also identified by the language (match). Note that this formula offers a normalized result in the interval [0,1], where 1 indicates a perfect match between the number of events identified by the experts and by the language.

(1) $$\mathrm{SI}M_{\mathrm{Ex}p_{\mathrm{Lang}}}=\frac{2*\#Ev_{\mathrm{Lang}-\mathrm{Exp}}}{\#Ev_{\mathrm{Exp}}+\#Ev_{\mathrm{Lang}}}$$

Looking at all 200 time series, there is found to be a close match between the experts and proposed language, as shown by the mean similarity (close to 96%) between the experts and language (Table 1).

The aim of the validation reported in Table 1 was to illustrate the match between events identified by the proposed technique and specified by the domain experts. With regard to language expressiveness, the experts who used the language did not pinpoint any weaknesses at all regarding missing elements or it being hard to use, etc.

Having validated the event identification procedure, the events were analysed statistically, taking into account the number of events of each class in the time series and the mean values of their attributes (Table 2).

Note that the above data are taken from a preliminary descriptive study and should not be construed as being illustrative of the final model, which is much more representative of the data sets and is, as explained in Section 4.5, output according to a much more sophisticated logic.

• Stabilometry

The stabilometric data used were from a total of 33 elite sportspeople, of which 15 were professional basketball players and 18 were elite ice-skaters. The studies focused on the US test, a test that provides interesting balance-related information. The events of interest occur when patients lose their balance and step on the platform (see Section 3.2). This test has four trials that are each repeated three times during a stabilometric examination. Therefore, we had access to a total of 33(subjects)*4(trials)*3(repetitions) = 396 time series.

We repeated the validation procedure on the 33 sportspeople. First, we applied the time series event identification method and compared the results with the events discovered by the experts using Eq. (1). The results are shown in Table 3, revealing a match greater than 98%.

Having validated the event identification procedure, the events were analysed statistically, taking into account the number events of each class in the time series and the mean values of their attributes (Table 4).

### 4.5 Data mining

The next step after transforming and reducing data is to apply data mining techniques to discover useful models. There are a great many possible time series data mining tasks, ranging from time series value prediction to time series classification. In the event of domains without special events (where, in principle, the whole time series is of equal interest), more conventional techniques based on feature set processing (k-means, K-NN, neural networks, etc.) can be applied. However, when time series contain events (as is the case of the research described in this paper), more made-to-measure alternatives have to be found. In this case, the techniques proposed for this purpose were:

■ A method for **comparing two patients** in order to output a measure of similarity between the two [68]. This similarity measure indicates how alike patients are or how a patient evolves over time. It is the baseline for solving other problems like outlier detection or reference model generation. The proposed method for comparing individuals is based on a comparison of the conceptual data models of the two subjects. This method is an algorithm for **comparing two time series**[62] based on the similarity of the events identified and characterized in both series.
■ The above comparison method as the starting point for a method for **generating reference models** from two or more patients [68]. The structure of the resulting reference model is again specified by the respective domain conceptual model. The resulting model should identify the elements common to all the subjects at each level of the conceptual model. Note that the algorithm for **generating reference models for time series** based on the cluster analysis of events [63], [64] using **clustering** techniques is the main part of this method. This method aims to pinpoint the events that are often found in the time series of the respective patients. These frequent events are the ones that best characterize the group of time series and, therefore, are built into the final reference model.
  In order to assure that outliers do not distort the resulting reference models, the reference model generation method also includes an **outlier detection and filtering** algorithm [68]. The outlier detection method is based on four criteria. These criteria are designed to emulate how human beings analysing clusters of objects identify outliers within a set of objects. This has an advantage over other clustering-based outlier detection techniques that are founded on a purely numerical analysis of clusters.

All the proposed algorithms were devised such that experts had to define the least possible number of input parameters, as physicians are not at all happy about rating these parameters with which they are mostly unfamiliar.

The above contributions are combined to solve the problem of **classification of individuals** (represented by their time series). Classification can be considered as a tool with many potential uses in the medical domain: diagnosis, early-age sports talent recruitment, study of patient evolution, etc.

The process of classifying individuals is based on a strategy combining the use of the method of comparing two patients and a method for generating reference models from a set of patients. The strategy followed to classify patients is as follows:

I: Generate, for each class *C*_*i*_ (*i* = 1, 2,…, *K*), a reference model (*M*_*i*_) from a training set of individuals.
II: Compare the new patient to be classified (*P*_*NEW*_) with each previously generated reference model *M*_*i*_ (*i* = 1, 2,…, *K*).
III: Select the class *C*_*j*_ whose reference model *M*_*j*_ is most similar to the new patient *P*_*NEW*_ such that *C*_*j*_ = *C*_*i*_ | similarity(*P*_*NEW*_,M_j_) = max(similarity(*P*_*NEW*_,*M*_*i*_)) ∀* i* = 1, 2,…, *K*.

**Fig. 9:**
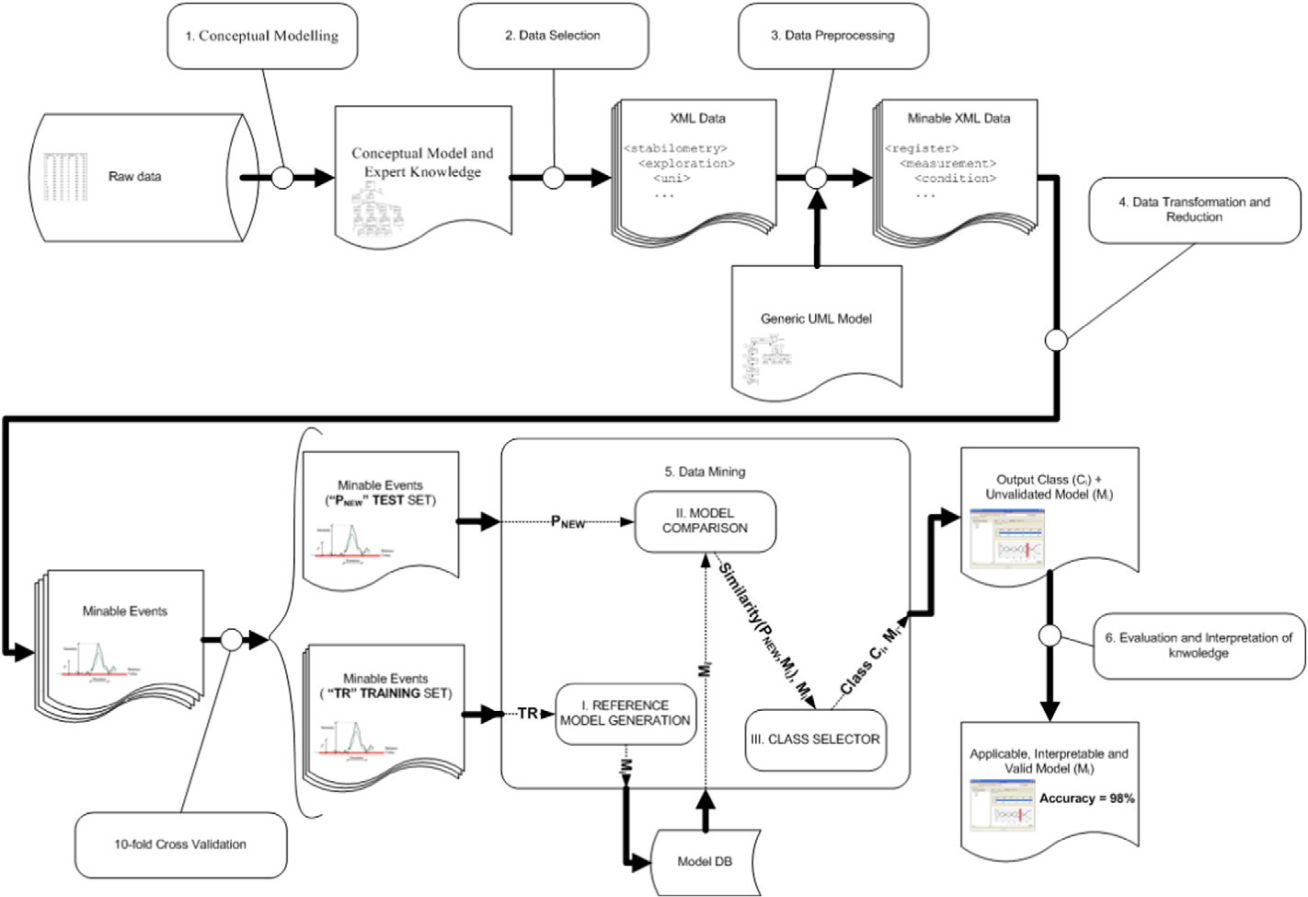
Overview of the process enacted in the case study (aligned with the KDD process).

The entire process described above is illustrated in the sequence diagram shown in Fig. 9, highlighting the different techniques applied in each phase of the process. Fig. 9 shows the original data source, the different intermediate products and steps of the process, and the discovered knowledge (valid model output after the interpretation/evaluation phase described in Section 4.6). The data mining phase enacts the above strategy consisting of: i) creating reference models, ii) comparing the element to be classified with each model, and iii) outputting the class depending on its similarities to each model.

The results of applying the proposed techniques in order to classify individuals have been satisfactory, as shown in Section 4.6. Before classification, it is necessary, as mentioned above, to filter out outliers. The outlier detection process is reported below.

• EEG

The outlier detection method was applied and evaluated based on the events identified previously using the event definition language. The time series comparison technique was applied to perform pairwise comparison on data from different patients This produced a similarity matrix for each pair of individuals. The outlier detection algorithm was then run on this matrix. This algorithm returns a list of the outlier individuals from the input matrix. On the other hand, the experts consulted in our research were asked to use conventional techniques to identify the individuals that they considered to be outliers. Table 5 shows the confusion matrix comparing the method and expert criteria.

Different indicators (precision, recall, specificity and accuracy) were calculated based on the above confusion matrix according to the formulae specified below.

**Table:** 

		Predicted Label	
	Positive	Negative	
Known label	Positive	True Positive (TP)	False Negative (FN)
	Negative	False Positive (FP)	True Negative (TN)

Precision = TP / (TP + FP)

Recall = TP / (TP + FN)

Specificity = TN / (TN + FP)

Accuracy = (TP + TN) / (TP + TN + FP + FN)

The values of the above indicators for the confusion matrix reported in Table 5 are shown in Table 6. In particular, the accuracy of the above results for the outlier detection method that we propose is 98%.

• Stabilometry

The time series outlier detection method was again applied as explained above. Table 7 illustrates the confusion matrix highlighting the comparison between the method and the experts.

The outlier detection performance indicators calculated based on this matrix are shown in Table 8. Worthy of special note is the overall accuracy value of 98.5%.

### 4.6 Evaluation/interpretation of discovered knowledge

#### 4.6.1 Evaluation

Domain experts were used to evaluate most of the proposed data mining techniques. In this case, the models yielded by applying the proposed techniques were compared against those generated by experts for validation purposes. This poses problems of differing criteria and subjectivity because it requires the participation of more than one expert.

• EEG

After filtering out the outliers, the remainder (individuals) were used to evaluate the classification method based on the generation of reference models against which the individuals to be classified are compared. To evaluate the mechanism, a series of experiments were run using the 10-fold cross-validation technique. This is a particular case of k-fold cross-validation, a clearly defined standard technique for validating classification techniques. The goal of this evaluation is to determine the quality of the classifications using the proposed techniques in terms of accuracy. The accuracy of a classifier *CF* is its probability of correctly classifying a randomly selected instance <* P*_*NEW*_,*C*_*i*_ >, i.e., accuracy = Pr (*CF*(*P*_*NEW*_) = *C*_*i*_) [74].

First, we generated two reference models, one for each class (*M*_*healthy*_ and *M*_*epileptic*_). In this field, healthy patients could be viewed as a control group. The first model (*M*_*healthy*_) was created from a training set composed of 90% of time series of the set of healthy patients (*A*). The other 10% of healthy patients were part of the test set. The second model (*M*_*epileptic*_) was generated from a training set composed of 90% of the time series in the epileptic patient set (*E*). The other 10% of patients were part of the test set. The patients in the test sets were chosen randomly.

Both generated models were evaluated to check whether *M*_*healthy*_ properly represents the group of healthy patients and *M*_*epileptic*_ is representative of the group of epileptic patients. To do this, we classified the individuals in the test sets according to their similarity to the two generated models (this similarity value was determined using the time series comparison method). This entire process was repeated 10 times, varying the training and test sets.

Table 9 reports a comparison of the results of classifying individuals from sets *A* (healthy) and E (epileptics) using the proposed knowledge discovery techniques, the AFINN system (a fuzzy neural network) and a multilayer perceptron. The best result for the above neural networks was using three layers, with three neurons in the input layer, one in the output layer and two in the middle layer. We used a classical sigmoid activation function and conducted backpropagation learning using the mean square error as a measure of total cost. The proposed approach was compared with these neural networks because they were familiar to us, as members of a research consortium partnered by other institutions that is applying and evaluating traditional neural network classification techniques on different data sets. The proposal was found to outperform neural network techniques.

Table 10 shows the events present in the two models built using the proposed techniques and their characteristics.

From the medical viewpoint, the reference models output by our proposal are, according to the above results, a promising option for epilepsy diagnosis from electroencephalographic examinations. With a classification accuracy greater than 99.8%, the proposed method is capable of correctly classifying patients suffering from epilepsy based on their EEG time series. Note that the proposed method and the resulting models are designed not as a medical diagnosis tool but rather as a medical decision-making aid.

• **Stabilometry**

After filtering out the outlier elements, a classification process was again enacted with the two problem classes (*M*_*basketball*_ and *M*_*skating*_). This process was performed using the same validation technique (10-fold cross validation). The results are shown in Table 11, where the classification accuracy for our method is greater than 99%, illustrating that our method outperforms the other analysed methods.

Table 12 shows the events present in the two models built using the proposed techniques and their characteristics.

From the medical viewpoint, these models reveal that balance is a variable related to the practised sport. In this case, there is a 99% likelihood of sportspeople being classified in their respective sport. These and other possible models for other sports have potential in the field of sports medicine, as balance (especially of young athletes) can help to classify sportspeople within the discipline for which they are best suited according to their postural control. This would help to point young sportspeople in the direction of the disciplines at which they are most likely to be proficient during early-age sports talent recruitment and possibly increase their future success as professional athletes.

In actual fact, both models in this classification problem represent sports disciplines, and there is no control group of non-athletes. This approach was taken because the key applicability of this method is to select the sports discipline for which each talented young athlete is best suited (the individuals are presumed and known to have potential as elite athletes).

#### 4.6.2 Interpretation

A fundamental design premise of the proposed techniques was that the resulting models should be easily interpretable by the respective type of expert user. Additionally, it was decided to use graphical elements (especially time series and their events) at all times for the purpose of ease of interpretation by specialists.

The stereotyped conceptual data model shown in Fig. 5 was a great help in this respect. For example, the result of the comparison of the stabilometric data of two individuals is a tree with the same structure as illustrated in Fig. 5. The tree is annotated with the similarity among the individuals at each level of the tree. The physician can browse the tree to study the similarities and differences between the two individuals under comparison. The result of the outlier detection process was a list of outlying values, sorted in top-down order.

**Fig. 10:**
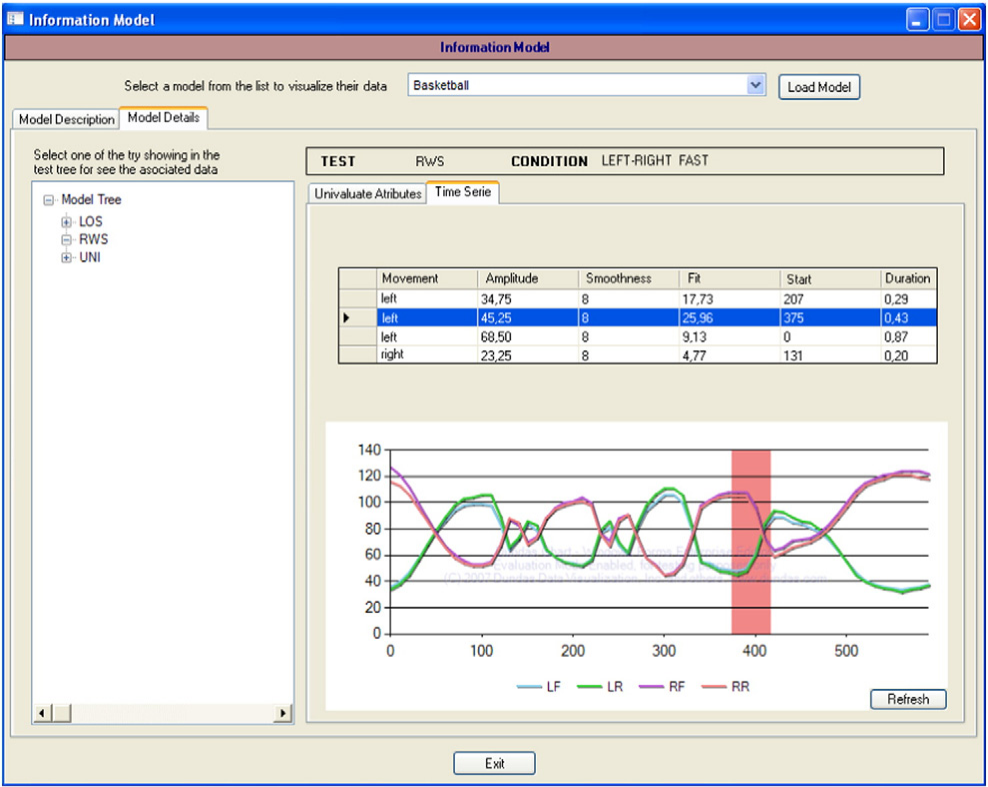
Model interpretation interface.

On the other hand, the generation of reference models results in an archetypal patient that represents a patient group. Using the proposed standard notation (see Fig. 5 for the stabilometric domain), the archetype has the same structure as any patient. This makes the model a lot easier for the medical expert to understand. Fig. 10 includes a sample screenshot of the application developed for this purpose, where the user can select the model (top), navigate the tree (left) and visualize the models as both a data table and chart (centre).

Medical experts are dynamic professionals who are always on the go. They have to travel from one institution to another, visit patients at home or athletes at training facilities, etc. Therefore, not only do the models have to be displayed by the application, but they also have to be exportable to manageable and printable formats (models displayed in Fig. 10 can also be exported to PDF).

## 5 Case Study Discussion

This section aims to discuss the different issues related to the reported case study and the techniques used. These issues are as follows.

### 5.1 a) General comments and lessons learned

The design of the proposed techniques was a troublesome process beset with complications that had to be addressed. One of the main handicaps was the shortage or temporary unavailability of experts in the reference domains, especially stabilometry, which is a relatively new discipline. The project would have failed if it had had a demanding schedule for deliverables and milestones. Therefore, one lesson learned is that, when dealing with medical specialists, the schedule has to be flexible.

Despite the difficulties, the medical specialists participated actively in the case study and took a lot of interest in the final results. It is true that, in many cases, physicians are not happy with the resulting models, when they are based only on historical cases. This case study, however, relied on expert knowledge (in order to define the events, which are the basis of the subsequent analysis), as well as on historical cases,.

We soon learned that medical data are very sensitive data whose acquisition is governed by sometimes very slow protocols. Additionally, there were very often not enough samples because of the complexity of the medical tests and the need to gain the patients' consent to use the medical data.

### 5.2 b) Comparison with other techniques

The case study found that the proposed approach outperforms other (neural network-based) methods that had been used with the respective test data in previous projects. Apart from improved predictability, the proposal has a sizeable added value compared to neural networks, as it shows the resulting models in a manner that is easy to interpret and justify.

As mentioned in Section 2, apart from the neural network-based approaches, the literature also describes special-purpose techniques for classifying time series. They include techniques based on the *k*-nearest neighbour algorithm. It is usual practice in this approach to use a measure of distance based on end-to-end differences among the series. The experiments described in the case study were repeated using this approach, and resulting accuracy rates were close to 50%. This algorithm behaves like a random classification system. This is because the analysis covers parts of the time series that are potentially of no interest to experts which are not filtered out. On the other hand, the *Shapelets*-based technique has the drawback of generating a single segment representing each class. This segment does not necessarily match any fragment of interest to the expert. Additionally, a reference model is generally composed of several representative segments (events) that are not necessarily adjacent. On this ground, the average accuracy of this proposal in the experiments conducted on our data was at most 62%.

It is true that, in all the above cases, the final result of our case study (an end-to-end process) was compared with the results of specific (independent) classification techniques because literature review failed to show up any proposals applying an end-to-end process (from the raw data to knowledge) on data from time series with events.

### 5.3 c) Proposal applicability

This paper described a case study applying data mining with time series containing events. Of course, this is a case study confined to two domains. However, the positive results hold out some promise for applying this proposal to other branches of medicine.

The only technique applicability condition is that the information of interest should be concentrated in certain regions of interest (events) of the time series to be analysed.

Event definition is the only part of the proposal that is domain dependent and requires expert participation. Event definition is easy to perform thanks to the proposed language, which is very like natural language and is very intuitive for experts, as evidenced by the experience of the medical experts that participated in this research. The other techniques are completely domain independent.

As regards the number of events, the applied techniques appear to work well irrespective of the number of events (the analysed data contain some series with few or no events and others with a sizeable number of events). For the purposes of applying this proposal, there is no limitation with respect to time series periodicity. The proposed approach identifies the events, irrespective of their periodicity, according to the conditions defined by experts for the purposes of characterization. A characteristic indicating the number of times that each event occurs in a periodic time series might be added in order to supplement the events.

Accordingly, our proposal is applicable to many areas, both inside and outside the field of medicine. In the medical domain, it could be applied to another type of times series like, for example, electrocardiograms, which contain periodic events.

### 5.4 d) Deployment of described techniques

As mentioned above, the proposal applied to this case study can be extended to other branches of medicine where time series with events are of special importance. The procedure would be as follows:

1. Thoroughly research the domain and data (especially the time series and their events) by means of interviews with experts and based on conceptual data modelling.
2. Select the data set to be used and arrange the data in XML files.
3. Reduce any noise and deal with missing values as explained in order to then automatically convert the specified XML data into XML data that conform to the standard UML pattern defined here.
4. Define the event types using the language designed for the purpose.
5. Apply the data mining techniques to output reference models of the classes to be studied (after removing outliers using the proposed method).
6. Compare the element to be classified with existing models to determine its class.
7. Based on the above results (repeated for each element), output model quality indicators (evaluation).
8. If the model quality indicators are good (typically accuracy is above a particular threshold), interpret and apply the above models.

Of course, the last step will involve implementing the respective techniques separately, in principle, albeit with the ultimate aim of building more comprehensive medical decision support system that integrates all the techniques. C# and the NetBeans 8.1 development environment were used in the reported the case study. Future integration requires exploration of which would be the best strategy to follow, that is, whether to use these or other technologies to further the above integration.

## 6 Conclusions and Future Lines

Iconographic time series, like electroencephalographic, stabilometric, electrocardiographic, etc., are increasingly common in medicine. This paper presented a number of specific knowledge discovery techniques applied on this type of time series from patient EHRs. Throughout this paper, we reported two empirical applications of the proposed techniques on data from the stabilometry and EEG domains throughout the different stages of the KDD process: from data comprehension, through data mining, to discovered knowledge interpretation and evaluation.

This paper, which reports the results and experience gained as a result of these case studies, aims to convey this knowledge to other researchers planning to use temporal data in the respective branches of medicine.

The experiments revealed that a surprising amount of useful knowledge can be gathered from this type of structures. The two specific examples reported in this paper show that is possible to discover knowledge from EHR-derived time series that is useful for medical experts. While medicine is possibly one of the richest domains for data mining engineers, it is definitely the toughest. To overcome this, we think that the scientific community needs to address the following challenges:

1. The design of tools to automate some resource-consuming time series analysis tasks, such as preparation.
2. The proposal of representation models capable of capturing all the singularities, heterogeneity and structural complexity of medical time series.
3. The specification of secure models for medical time series storage and publication with the aim of increasing efficient data reuse and processing.
4. The implementation of time series visual support tools for medical specialists.

The main future lines that we intend to address are as follows:

1. A tool for visually defining events in time series which is currently a text-based process
  Research is now centring on a visual tool to enable experts to identify events in time series. This tool is composed of an interface that displays graphs of different time series for experts. Experts can use the mouse to point to the regions that they consider of interest (events). This proposal infers the conditions that the identified regions meet (analyses aspects such as time series maximums or minimums, changes of trend, etc.), which it maps to the event definition language code. Clearly, this tool acts an intermediary between the experts and the event definition language (which is rather complex for experts who have no experience in using programming languages or similar).
2. A visual tool for managing panels of experts and applying the Delphi method [69], [75]
  The tool described above is rounded out by another tool that considers the opinion of several rather than just one medical expert. Expert collaboration via the Delphi method renders the gold-standard annotation scheme more objective, and the events more accurate. However, expert availability is low, for which reason we are working on a tool capable of applying the Delphi method remotely and asynchronously. It is actually a web application that manages the different rounds of the Delphi method by sending out warnings and reminders to the email addresses of the participating experts according to an established schedule. The preliminary results are satisfactory with respect to both lines of research.
3. Extend the comparison beyond neural networks
  The possibility of examining whether the results of applying the techniques described in this case study are better than other data mining techniques that were not devised for purely classification purposes, like, for example, logistic regression is worth considering.
4. Study data computing performance
  This paper reported an evaluation of the proposed techniques in terms of effectiveness and usefulness (in this case, by means of classification accuracy). Although the response times of the techniques applied are viewed by the experts as being acceptable, future research should specifically examine the computational complexity of the above techniques depending on data characteristics (time series size and dimensionality, number of subjects, etc.). Public gold standard datasets should be used for this purpose.

## Figures and Tables

**Table 1:** Overall results of applying the event definition language to the EEG domain.

#Series	#*Ev*_*Exp*_	#*Ev*_*Lang*_	#*Ev*_*Lang* − *Exp*_	***SIM***_***Exp***_***Lang***__
200	1446	1496	1412	0.959

**Table 2:** Statistical analysis of the events identified in the EEG domain.

Class	Avg(#Events)	Avg(Duration) (ms)	Avg(Amplitude)
*Epileptic*	9.47	195	78
*Healthy*	5.49	56	54

**Table 3:** Overall results of the application of the event definition language to the stabilometric domain.

#Series	#*Ev*_*Exp*_	#*Ev*_*Lang*_	#*Ev*_*Lang* − *Exp*_	***SIM***_***Exp***_***Lang***__
396	942	954	931	0.982

**Table 4:** Statistical analysis of events identified in the stabilometric domain.

Class	Avg(#Events)	Avg(Duration) (ms)	Avg(Intensity)
*Basketball*	3.37	754	107
*Skating*	1.45	346	83

**Table 5:** Confusion matrix for the application of the outlier detection method to the EEG domain.

		Language	
	**Outlier**	Yes	No
Experts	Yes	11	1
	No	3	185

**Table 6:** Overall results for the application of the outlier detection method to the EEG domain.

Indicator	Value
*Precision*	78.6
*Recall*	91.7
*Specificity*	98.4
*Accuracy*	98.0

**Table 7:** Confusion matrix for the application of the outlier detection method to the stabilometric domain.

		Language	
	**Outlier**	Yes	No
Experts	Yes	13	2
	No	4	377

**Table 8:** Overall results for the application of the outlier detection method to the stabilometric domain.

Indicator	Value
*Precision*	76.5
*Recall*	86.7
*Specificity*	99
*Accuracy*	98.5

**Table 9:** Comparison of the classification of patients by different methods in the EEG domain (accuracy)

Patient type	Knowledge discovery in time series	AFINN	Multilayer perceptron
*Epileptic*	99.86%	96.26%	96.61%
*Healthy*	98.11%	95.12%	93%

**Table 10:** Reference models built by our proposal for EEG.

Model	#Event	Duration (ms)	Amplitude	#Timestamp
*Epileptic*	1	56	74	345
	2	321	81	1022
	3	68	73	3429
	4	167	87	2879
	5	189	83	1758
	6	245	92	895
	7	76	101	2210
			
*Healthy*	1	89	45	1355
	2	145	32	3652
	3	110	37	345
	4	57	21	1384

**Table 11:** Comparison of the classification of patients by different methods in stabilometric domain (accuracy)

Patient type	Knowledge discovery in time series	AFINN	Multilayer perceptron
*Basketball*	99.4	98.8	97.7
*Skating*	99.1	98.1	97.1

**Table 12:** Reference models built by the proposed method for stabilometry.

Model	#Event	Duration (ms)	Intensity	#Timestamp
*Basketball*	1	854	103	345
	2	723	96	783
	3	1099	117	267
*Skating*	1	234	73	211
